# Definitive Chemoradiotherapy Versus Total Laryngectomy Followed by Postoperative Therapy in Selected T3–T4a N0–N1 Laryngeal Cancer: Comparative Outcomes

**DOI:** 10.3390/cancers18182966

**Published:** 2026-09-14

**Authors:** Ozlem Ozkaya Akagunduz, Tugce Bozkurt Vardar, Ecem Yigit, Gozde Yazici, Fethiye Odaci, Mervenur Bay, Kerem Ozturk, Mustafa Cengiz, Oguz Kuscu, Gokhan Ozyigit, Mustafa Esassolak

**Affiliations:** 1 Department of Radiation Oncology, Ege University Faculty of Medicine, 35100 Izmir, Türkiye; 2 Department of Radiation Oncology, Hacettepe University Faculty of Medicine, 06230 Ankara, Türkiye; 3 Department of Otorhinolaryngology, Ege University Faculty of Medicine, 35100 Izmir, Türkiye; 4 Department of Otorhinolaryngology, Hacettepe University Faculty of Medicine, 06230 Ankara, Türkiye

**Keywords:** laryngeal cancer, larynx preservation, definitive chemoradiotherapy, total laryngectomy, advanced laryngeal cancer, oncologic outcomes

## Abstract

**Background/Objectives**: Management of locally advanced laryngeal squamous cell carcinoma remains controversial. This study compared definitive chemoradiotherapy (CRT) with total laryngectomy followed by postoperative radiotherapy with or without chemotherapy (TL + PORT/CRT) in T3–T4aN0–N1 disease. **Methods**: This multicenter retrospective cohort included 311 patients treated between 2008 and 2025 at two centers: 160 received definitive CRT and 151 underwent TL + PORT/CRT. Survival was estimated using Kaplan–Meier and Cox regression analyses. Propensity score matching (PSM) and inverse probability of treatment weighting (IPTW) were used as adjusted analyses. The primary endpoint was disease-specific survival (DSS). **Results**: Median follow-up was 98 months. In the unmatched cohort, 5- and 10-year DSS rates were 92.1% and 92.1% after CRT versus 93.7% and 83.5% after TL + PORT/CRT (*p* = 0.351), while 5-year local control favored TL + PORT/CRT (98% vs. 79%; *p* < 0.001). After PSM (59 patients per group), cluster-robust Cox analysis showed inferior overall survival (OS) after CRT (hazard ratio [HR], 2.08; 95% confidence interval [CI], 1.12–3.86; *p* = 0.021) and inferior local control (HR, 12.83; 95% CI, 3.75–43.87; *p* < 0.001). In the 2015–2025 intensity–modulated radiotherapy (IMRT)-restricted analysis, OS also favored TL + PORT/CRT (HR, 3.64; 95% CI, 1.09–12.22; *p* = 0.036), whereas full-cohort IPTW showed no significant OS difference (HR, 0.93; 95% CI, 0.64–1.36; *p* = 0.715). Functional laryngeal preservation was achieved in 126/160 patients (78.8%) after CRT. **Conclusions**: Definitive CRT was associated with inferior local control, while DSS remained comparable. OS estimates varied across adjusted analyses and did not support a stable treatment-specific survival effect. Definitive CRT remains an organ-preservation option in appropriately selected patients.

## Simple Summary

For selected patients with advanced laryngeal cancer, treatment decisions must balance cancer control with preservation of breathing, swallowing, and voice function. Total laryngectomy provides strong local tumor control but permanently removes the larynx, whereas definitive chemoradiotherapy may preserve the organ but carries a risk of local recurrence. In this multicenter retrospective study, we compared definitive chemoradiotherapy with total laryngectomy followed by postoperative treatment in patients with T3–T4a laryngeal cancer and limited nodal disease. Although surgery provided better local control, disease-specific survival was similar between treatment groups, and adjusted analyses did not demonstrate a consistent overall-survival advantage for either treatment strategy. Salvage surgery was effective in many patients who developed local recurrence after chemoradiotherapy. These findings suggest that definitive chemoradiotherapy may remain a reasonable organ-preservation strategy in carefully selected patients when close follow-up and salvage surgery are available.

## 1. Introduction

The larynx is a critical organ in the head and neck region, playing a central role in maintaining airway patency, phonation, and swallowing function. Therefore, although achieving oncologic cure remains the primary goal of treatment for laryngeal cancer, the preservation of laryngeal function and the maintenance of quality of life have become integral components of modern oncologic management [1,2,3,4].

Accordingly, since the early 1990s, organ-preservation strategies have been developed as alternatives to total laryngectomy. Based on the results of landmark trials conducted by the Veterans Affairs (VA) Laryngeal Cancer Study Group, the European Organisation for Research and Treatment of Cancer (EORTC), the Radiation Therapy Oncology Group (RTOG), and the Groupe Oncologie Radiothérapie Tête Et Cou (GORTEC), definitive radiotherapy (RT) and concurrent chemoradiotherapy (CRT) have become widely accepted treatment approaches for selected patients with locally advanced laryngeal cancer [5,6,7,8]. These studies demonstrated that laryngeal preservation could be achieved without compromising survival in appropriately selected patients, leading to a paradigm shift in head and neck oncology and establishing organ-preserving approaches as standard treatment options.

However, following the widespread adoption of organ-preservation strategies, several retrospective population-based studies have suggested that selected patients with locally advanced (T3–T4a) laryngeal cancer, particularly those with advanced T stage or unfavorable disease characteristics, may experience inferior oncologic outcomes after definitive RT/CRT compared with surgery-based approaches, including lower overall survival, higher rates of local failure, and increased need for salvage procedures [9,10,11]. In particular, large-scale analyses using the National Cancer Database (NCDB) have demonstrated that the increasing adoption of organ-preservation strategies has coincided with declining survival trends among patients with T3 laryngeal cancer. Furthermore, population-based studies have suggested that primary total laryngectomy may provide more consistent oncologic outcomes in selected patients with T4 tumors, particularly those with cartilage invasion or impaired pretreatment laryngeal function [12,13,14,15].

Nevertheless, it is important to recognize that many of these studies were conducted using imaging modalities and radiotherapy techniques that do not reflect current clinical practice. During the era of conventional two-dimensional (2D) radiotherapy planning, limitations in target volume delineation and inadequate sparing of normal tissues contributed to substantial functional morbidity, including severe dysphagia, aspiration, long-term tracheostomy dependence, and percutaneous endoscopic gastrostomy (PEG) dependence [16,17,18,19].

In the modern era, advances in functional and metabolic imaging, together with the implementation of intensity-modulated radiotherapy (IMRT), volumetric-modulated arc therapy (VMAT), and adaptive radiotherapy techniques, have enabled more accurate target delineation and improved protection of critical normal structures [20,21,22,23]. These technological advances may limit the applicability of historical treatment outcomes to contemporary clinical practice and highlight the need to reevaluate organ-preservation strategies in the context of modern treatment approaches [24,25].

In this context, redefining the role of definitive chemoradiotherapy in patients with T3–T4a laryngeal cancer and limited nodal burden (N0–N1) is clinically relevant, particularly regarding local control, overall survival, and functional laryngeal preservation. Therefore, this retrospective cohort study aimed to compare the oncologic and functional outcomes of patients treated with definitive chemoradiotherapy with those of patients undergoing surgery followed by adjuvant radiotherapy or chemoradiotherapy, in order to clarify the role of organ-preservation strategies in contemporary clinical practice.

## 2. Materials and Methods

### 2.1. Study Design and Patient Population

This multicenter retrospective cohort study included patients with locally advanced laryngeal squamous cell carcinoma treated at the Departments of Radiation Oncology at Ege University Faculty of Medicine and Hacettepe University Faculty of Medicine between 2008 and 2025. Patients with T3–T4a and N0–N1 disease who underwent curative-intent treatment with either definitive chemoradiotherapy (CRT) or total laryngectomy (TL) followed by postoperative radiotherapy (PORT) or postoperative chemoradiotherapy were included in the analysis.

Clinical, treatment, pathological, radiological, and follow-up data were retrospectively collected from institutional medical records, radiation oncology treatment databases, pathology reports, imaging archives, and follow-up records.

Eligible patients were required to have histologically confirmed laryngeal squamous cell carcinoma, a primary tumor stage of T3–T4a, nodal status of N0–N1, no evidence of distant metastasis at diagnosis, and completion of curative-intent treatment with either definitive CRT or TL followed by PORT or postoperative CRT. Patients were required to have sufficient clinical, treatment, and follow-up information for outcome analysis.

Patients were excluded if they had distant metastasis at diagnosis, non-squamous histology, recurrent disease at presentation, previous radiotherapy to the head and neck region, palliative treatment intent, or insufficient data for survival analysis.

### 2.2. Pretreatment Evaluation and Treatment

Pretreatment evaluation included clinical examination, endoscopic assessment of the larynx and upper aerodigestive tract, histopathological confirmation by biopsy, and radiological evaluation of the primary tumor and cervical lymph nodes. Imaging assessment consisted of magnetic resonance imaging (MRI) and/or computed tomography (CT), with positron emission tomography/computed tomography (PET/CT) performed when clinically indicated and available [26,27,28]. Treatment decisions were made according to multidisciplinary tumor board recommendations at each institution. Tumor staging was assigned according to the 8th edition of the American Joint Committee on Cancer (AJCC) staging system [29]. Patients treated before the implementation of the AJCC 8th edition were retrospectively restaged using available clinical examination findings, endoscopic assessments, radiological imaging, pathology reports, and operative records, as applicable. A total of 166 patients (53.4%) were retrospectively restaged, including 60 of 160 patients (37.5%) in the definitive CRT group and 106 of 151 patients (70.2%) in the TL + PORT group. In non-operated patients, clinical extranodal extension was assessed on pretreatment imaging based on radiological evidence of extension beyond the nodal capsule and/or infiltration of adjacent soft tissues. Accordingly, all T4 tumors included in the cohort were classified as T4a. Baseline tumor characteristics, including laryngeal cartilage invasion and vocal cord fixation, were assessed based on pretreatment clinical examination, endoscopic findings, and radiological imaging and were recorded from the patients’ medical records. Baseline laryngeal function was also assessed clinically before treatment. Patients presenting with bilateral vocal cord fixation and markedly impaired laryngeal function at baseline were considered unsuitable for an organ-preservation approach and were therefore treated with total laryngectomy. Because these variables were not consistently available in a standardized, patient-level format across the retrospective cohort, they were not included in the propensity score model.

Patients were categorized into two treatment groups according to the initial treatment strategy: definitive CRT or TL followed by PORT with or without chemotherapy.

The definitive CRT group included patients treated with curative-intent concurrent chemoradiotherapy. Radiotherapy was delivered using intensity-modulated radiotherapy (IMRT). The median total radiotherapy dose was 69.7 Gy (range, 64–71 Gy). Concurrent systemic therapy was administered in the majority of patients, predominantly using cisplatin-based regimens. The definitive CRT cohort included heterogeneous systemic therapy approaches, including induction followed by concurrent treatment and different concurrent agents, reflecting changes in clinical practice and patient-specific treatment selection over the study period.

The surgical treatment group consisted of patients who underwent TL followed by postoperative radiotherapy or postoperative chemoradiotherapy. Postoperative radiotherapy was delivered using either three-dimensional conformal radiotherapy (3D-CRT) or IMRT. The median postoperative radiotherapy dose was 61.3 Gy (range, 60–66 Gy). Concurrent chemotherapy was administered in 77 patients based on adverse pathological features and institutional treatment decisions.

Pathological variables, including lymphovascular space invasion (LVSI), perineural invasion (PNI), and surgical margin status, were evaluated only in patients undergoing surgical treatment.

### 2.3. Data Collection and Variables

Patient, tumor, treatment-related, and pathological characteristics were retrospectively collected. Demographic and clinical variables included age, sex, smoking history, alcohol consumption, primary tumor subsite, T stage, N stage, and overall stage. Treatment-related variables included treatment modality, chemotherapy administration, chemotherapy regimen and timing, radiotherapy technique, and total radiotherapy dose.

Primary tumor subsite was classified according to the predominant site of origin as supraglottic, glottic, or transglottic. Twenty-four tumors initially recorded as subglottic in one institutional dataset were re-reviewed and found to represent glottic–subglottic tumors in which a distinct subglottic primary epicenter could not be reliably established. These cases were therefore reclassified within the glottic group, and the separate subglottic category was removed from the analysis.

Smoking history was categorized as ≤30 pack-years or >30 pack-years. For prognostic analyses, cumulative cisplatin dose was evaluated only among patients who received cisplatin and was categorized as <200 mg/m^2^ or ≥200 mg/m^2^. Tumor size was determined using the maximum primary tumor dimension on pretreatment cross-sectional imaging in patients treated with definitive CRT, whereas the pathological tumor size reported in the surgical specimen was used for patients undergoing total laryngectomy. Tumor size was categorized as ≤2.5 cm or >2.5 cm for survival analyses, using the median measurable tumor size in the study cohort as the cutoff. Treatment modality was classified as definitive CRT versus TL followed by PORT with or without chemotherapy.

### 2.4. Outcomes and Follow-Up

The primary endpoint was disease-specific survival (DSS), defined as the interval from the date of diagnosis to death attributable to laryngeal cancer. Patients who were alive at the last follow-up or who died from causes unrelated to laryngeal cancer were censored at the date of last follow-up.

Functional laryngeal preservation was assessed in the entire definitive CRT cohort (*n* = 160) and was defined as survival with the larynx in situ, absence of local recurrence, no permanent tracheostomy, and no long-term feeding-tube dependence at the last available assessment. Patients who developed local recurrence were not considered to have achieved functional laryngeal preservation, irrespective of whether salvage laryngectomy was subsequently performed.

Secondary endpoints included overall survival (OS), local control (LC), regional control (RC), and distant metastasis-free survival (DMFS). OS was defined as the interval from diagnosis to death from any cause. LC, RC, and DMFS were defined as the intervals from diagnosis to the first occurrence of local recurrence at the primary tumor site, regional recurrence in the cervical lymph nodes, or distant metastasis, respectively. Local recurrence was defined as disease developing after a documented complete response to primary treatment; persistent residual disease at the initial post-treatment assessment was not classified as recurrence. All local recurrences were histopathologically confirmed. Laryngectomy-free survival (LFS) was defined as the time from the date of diagnosis to the first occurrence of total laryngectomy or death from any cause, whichever occurred first. Patients who were alive without laryngectomy at the last available follow-up were censored at that time. Patients with unknown final vital status were censored at their last known follow-up. Patients without the corresponding event were censored at the date of the last follow-up.

Follow-up was performed according to institutional protocols using a similar surveillance approach in both treatment groups. Patients were generally evaluated every 3 months during the first follow-up year, every 6 months thereafter, and annually during subsequent years. Follow-up assessments included clinical examination, endoscopic evaluation, and radiological imaging at regular intervals. The final follow-up date was February 2026. As this was a retrospective study, this date represents the cutoff for pre-existing routine clinical follow-up data and therefore preceded the date of ethics approval; research data extraction and analysis were performed after ethics approval.

### 2.5. Statistical Analysis

Descriptive statistics were reported as mean, standard deviation, median, range, frequency, and percentage, as appropriate. Survival outcomes were estimated using the Kaplan–Meier method, and median follow-up was calculated using the reverse Kaplan–Meier method. Univariable Cox proportional hazards regression was used to evaluate factors associated with local control, distant metastasis-free survival, and disease-specific survival. Parsimonious multivariable Cox models including treatment group, T stage, and age were fitted for OS and DSS. Hazard ratios (HRs) and 95% confidence intervals (CIs) were reported. Missing data were not imputed except in the inverse probability of treatment weighting (IPTW) analysis, for which multiple imputation was used. For propensity score matching, missing smoking-history and tumor-size values were retained as a separate “Unknown” category; therefore, all 311 patients were eligible for the propensity score model. Other analyses were performed using available complete data for the variables included in each analysis. This approach was used to avoid complete exclusion of patients with unavailable tumor-size measurements from propensity score estimation. However, because tumor-size missingness was strongly differential between treatment groups, the resulting matched cohort was considered potentially susceptible to selection related to measurement availability. In contrast, the IPTW analysis used multiple imputation for missing covariate information to retain all 311 patients. These two missing-data approaches are therefore not methodologically equivalent, and greater emphasis was placed on the full-cohort IPTW estimate when interpreting overall survival.

Propensity score matching (PSM) was performed in the entire cohort using age, sex, smoking history, T stage, N stage, and tumor size. Patients were matched 1:1 using nearest-neighbor matching with a caliper of 0.2 standard deviations of the logit of the propensity score, resulting in 59 patients per group. Covariate balance was assessed using absolute standardized mean differences (SMDs), with values <0.20 considered acceptable. To account for within-pair dependence in the propensity score-matched cohort, Cox proportional hazards models were additionally fitted using a cluster-robust variance estimator based on matched-pair identifiers. Because PSM retained only 118 of 311 patients and tumor-size missingness differed substantially between treatment groups, IPTW was used as the principal full-cohort adjusted sensitivity analysis. IPTW was performed using the same six covariates included in the propensity score model, with multiple imputation of missing covariate information to retain the entire cohort; covariate balance after weighting was assessed using absolute SMDs. The PSM analysis was therefore interpreted as a complementary, subset-specific sensitivity analysis.

A calendar-era sensitivity analysis was performed among patients treated between 2015 and 2025 who received IMRT in both treatment groups. The proportional hazards assumption was assessed using Schoenfeld residuals; when not satisfied, restricted mean survival time (RMST) up to 60 months was used as a complementary measure. Competing-risk analyses were performed for salvage laryngectomy, local recurrence, and distant metastasis, with death from any cause treated as a competing event. Cumulative incidence functions were estimated using a competing-risk framework and, for local recurrence and distant metastasis, the Aalen–Johansen method.

All statistical analyses were performed using IBM SPSS Statistics for Windows, Version 25.0 (IBM Corp., Armonk, NY, USA). R version 4.4.1 (R Foundation for Statistical Computing, Vienna, Austria; packages: survival and forestploter) was used for graphical visualization. All tests were two-sided, and *p* < 0.05 was considered statistically significant.

## 3. Results

### 3.1. Patient and Treatment Characteristics

**Figure 1:**
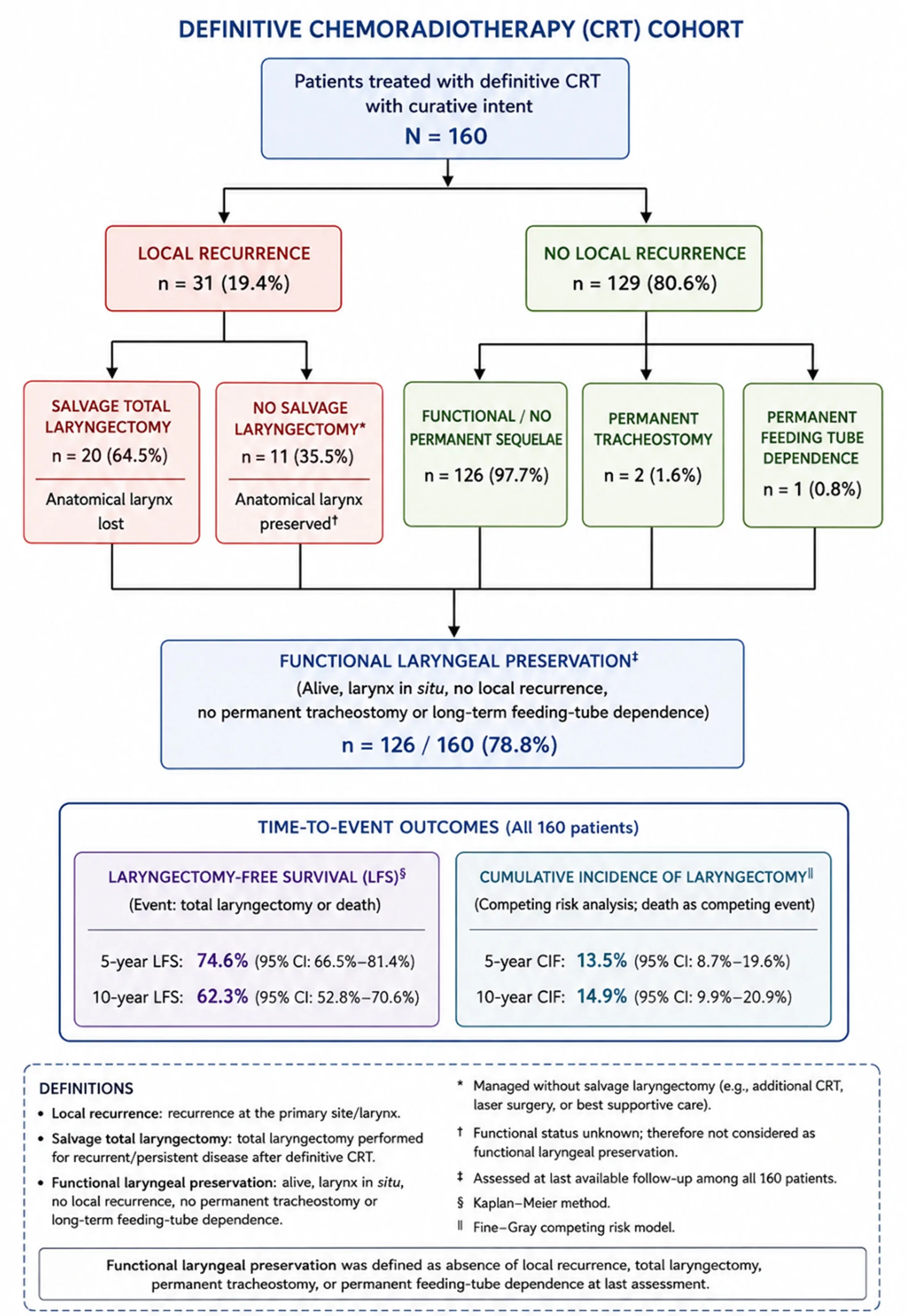
Outcomes in the definitive CRT cohort (*N* = 160) with exact denominators. Arrows indicate patient flow through local recurrence, salvage treatment, functional laryngeal preservation, and time-to-event analyses.

The revised patient flow diagram provides the exact denominators at each stage of follow-up, including local recurrence, salvage total laryngectomy, and assessment of functional laryngeal preservation (Figure 1).

A total of 322 patients were initially assessed for eligibility. Four patients were excluded because of non-squamous histology, five because adequate follow-up data were unavailable, and two because they did not complete the planned treatment, leaving 311 patients for the final analysis. Of these, 151 patients underwent total laryngectomy followed by postoperative radiotherapy (TL + PORT), whereas 160 patients received definitive chemoradiotherapy (CRT).

The median age of the entire cohort was 61 years, and 95% of patients were male. In the definitive CRT group, 81.3% of patients had T3 disease, whereas 70.9% of patients in the TL + PORT group had T4 disease. Approximately half of the patients in the TL + PORT group received concurrent adjuvant chemotherapy. In the definitive CRT group, weekly cisplatin was the most commonly administered concurrent chemotherapy regimen. All patients treated with definitive CRT received intensity-modulated radiotherapy (IMRT). Detailed patient and treatment characteristics are summarized in Table 1.

### 3.2. Survival and Disease Control Outcomes

The median follow-up duration estimated using the reverse Kaplan–Meier method was 98 months (95% CI, 86–110 months). When estimated separately by treatment group, the median follow-up was 119 months (95% CI, 112–130 months) in the TL + PORT group and 74 months (95% CI, 67–92 months) in the definitive CRT group. Patient accrual was distributed as follows: 92 patients were treated in 2008–2012, 97 in 2013–2017, 106 in 2018–2022, and 16 in 2023–2025. Thus, only 16 patients (5.1%) were treated during the most recent accrual period (2023–2025), and these patients contributed limited follow-up to the 10-year outcome estimates. The 5- and 10-year overall survival (OS) rates were 73.5% and 45.8%, respectively, in the TL + PORT group and 75.2% and 62.2%, respectively, in the definitive CRT group, with no statistically significant difference between the treatment groups (*p* = 0.088). For the entire cohort, the 5- and 10-year disease-specific survival (DSS) rates were 93% and 86%, respectively. The 5- and 10-year DSS rates were 93.7% (95% CI, 89.7–97.8%) and 83.5% (95% CI, 75.8–91.9%), respectively, in the TL + PORT group and 92.1% (95% CI, 87.7–96.7%) at both time points in the definitive CRT group. There was no statistically significant difference in DSS between the treatment groups (*p* = 0.351). In parsimonious multivariable Cox models including treatment group, T stage, and age, treatment modality was not independently associated with overall survival (HR, 0.92; 95% CI, 0.61–1.37; *p* = 0.670) or disease-specific survival (HR, 0.98; 95% CI, 0.44–2.18; *p* = 0.962). For overall survival, T4 disease and older age were independently associated with poorer survival.

**Figure 2:**
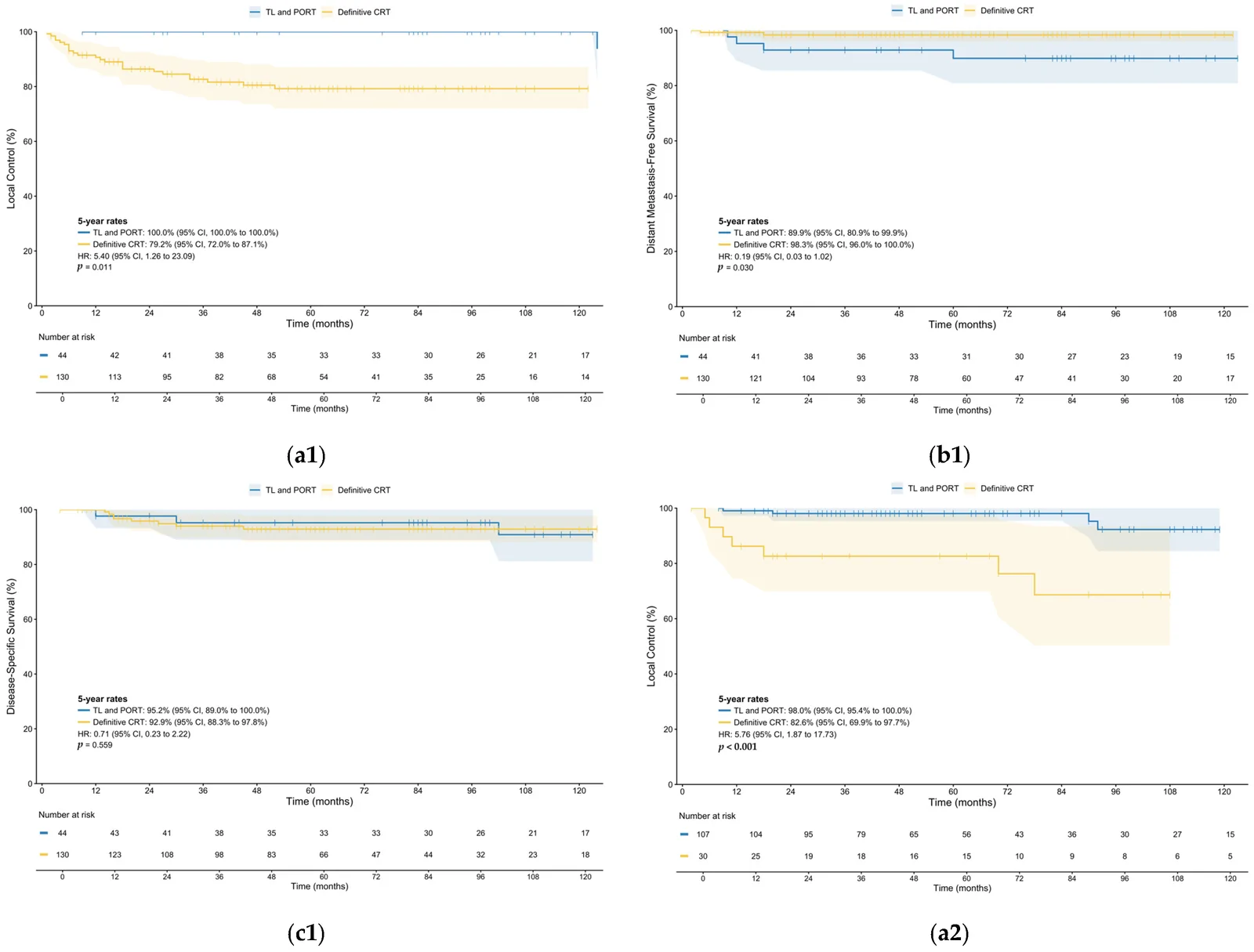

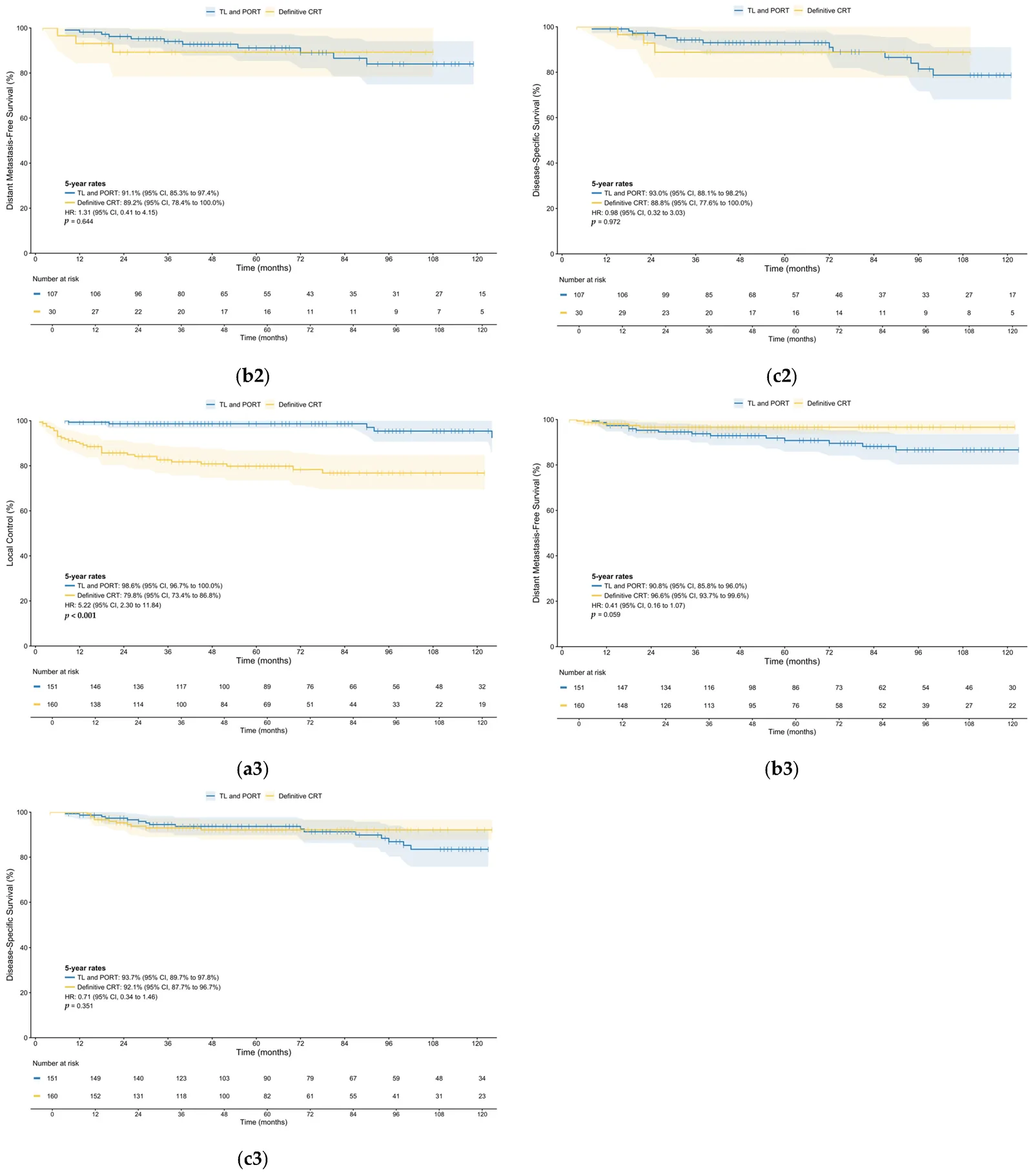
Comparison of oncologic outcomes between total laryngectomy with postoperative radiotherapy (TL + PORT) and definitive chemoradiotherapy (CRT): (**a1**) local control in T3 tumors; (**b1**) distant metastasis-free survival in T3 tumors; (**c1**) disease-specific survival in T3 tumors; (**a2**) local control in T4 tumors; (**b2**) distant metastasis-free survival in T4 tumors; (**c2**) disease-specific survival in T4 tumors; (**a3**) local control in the combined T3–T4 cohort; (**b3**) distant metastasis-free survival in the combined T3–T4 cohort; and (**c3**) disease-specific survival in the combined T3–T4 cohort.

A statistically significant difference was observed in local control (LC) between the two treatment groups. The 5- and 10-year LC rates were 98% and 95% in the TL + PORT group compared with 79% and 77% in the definitive CRT group, respectively (*p* < 0.001). Among patients treated with definitive CRT, the 5-year LC rates were 79.2% (95% CI, 72.0–87.1%) for T3 tumors and 82.6% (95% CI, 69.9–97.7%) for T4 tumors. Although the point estimate was numerically higher for T4 disease, this finding should be interpreted cautiously given the small number of T4 patients (*n* = 30) and the wide confidence interval. Figure 2 demonstrates the differences in local control, distant metastasis-free survival, and disease-specific survival according to T stage and treatment modality (TL + PORT versus definitive CRT).

In the calendar-era and IMRT-restricted sensitivity analysis, which included 124 patients treated with definitive CRT and 54 treated with TL + PORT, the median follow-up was 60 and 56 months, respectively, as estimated using the reverse Kaplan–Meier method. There were 21 overall survival events in the definitive CRT group and 3 in the TL + PORT group. Five-year OS was 83.5% (95% CI, 74.6–89.5%) after definitive CRT and 98.1% (95% CI, 87.1–99.7%) after TL + PORT (HR, 3.64; 95% CI, 1.09–12.22; *p* = 0.036), with 52 and 26 patients remaining at risk at 5 years, respectively. The corresponding 60-month RMSTs were 54.6 and 59.4 months. Local control remained inferior after definitive CRT, with 22 local recurrences compared with one after TL + PORT and 60-month RMSTs of 52.0 and 60.0 months, respectively. The single local recurrence in the TL + PORT group occurred at 92 months; therefore, no local recurrence occurred within the first 5 years, explaining the 100% 5-year LC estimate despite an estimable Cox hazard ratio over the complete follow-up period. Given the small number of overall survival events in the TL + PORT subgroup and the limited number of patients remaining at risk at 5 years, the OS estimate should be interpreted cautiously. These findings were consistent with the primary time-to-event analyses, particularly with respect to the difference in local disease control (Table 2; Supplementary Tables S1 and S2).

In competing-risk sensitivity analyses restricted to the same calendar-era and IMRT cohort, with death from any cause treated as a competing event, the 5-year cumulative incidence of local recurrence was 19.0% in the definitive CRT group and 0% in the TL + PORT group. The corresponding 5-year cumulative incidence of distant metastasis was 2.5% and 7.8%, respectively. These findings support the persistence of the difference in local disease control, while no consistent difference in distant metastatic control was observed.

Among the 160 patients receiving definitive CRT, local recurrence developed in 31 patients (19.4%). Salvage total laryngectomy was performed in 20 of these patients (64.5%), and five patients subsequently received re-irradiation. Disease recurrence after salvage surgery occurred in three patients. Two additional patients died from intercurrent causes. Thus, 15/20 (75%) patients who underwent salvage total laryngectomy were alive and disease-free at the last follow-up. Following salvage laryngectomy, the 5- and 10-year local control rates were 89% and 78%, respectively. The remaining 11 patients with local recurrence did not undergo salvage laryngectomy and were managed according to clinical circumstances.

No statistically significant difference was observed between treatment groups in terms of regional control (RC) (*p* > 0.05). Distant metastasis-free survival (DMFS) was numerically higher in the definitive CRT group than in the TL + PORT group, but the difference did not reach statistical significance. The 5- and 10-year DMFS rates were 90% and 87%, respectively, in the TL + PORT group, compared with 97% at both time points in the definitive CRT group (*p* = 0.059). Distant metastases developed in six patients in the definitive CRT group, all involving the lungs. In the TL + PORT group, distant metastases occurred in 15 patients. Pulmonary metastasis was observed in all cases, with concomitant bone and liver metastases identified in some patients.

Secondary primary malignancies developed in 12% of patients in both treatment groups. Lung cancer was the most common secondary malignancy, accounting for 68% of cases in the definitive CRT group and 88% of cases in the TL + PORT group. No treatment-related mortality was observed.

### 3.3. Propensity Score-Matched Analysis

To account for baseline differences between the treatment groups, propensity score matching was performed, yielding 59 patients in each group. After matching, all absolute standardized mean differences were <0.20, indicating good balance between the groups (Table 3). The substantial imbalance in T stage observed before matching was markedly reduced after matching (absolute SMD, 1.226 before matching vs. 0.068 after matching).

Comparison of patients retained in the propensity score–matched cohort with those who remained unmatched demonstrated substantial selection within both treatment groups. In the definitive CRT group, T4 disease was present in 42.4% of matched patients compared with 5.0% of unmatched patients, whereas unknown tumor size decreased from 54.5% among unmatched patients to 5.1% among matched patients. Conversely, in the TL + PORT group, T4 disease was present in 45.8% of matched patients compared with 87.0% of unmatched patients. Consistent with these differences, 5-year OS was 64.5% versus 81.7% in matched and unmatched definitive CRT patients, respectively, and 84.0% versus 63.5% in matched and unmatched TL + PORT patients, respectively. Baseline characteristics and crude outcomes of matched and unmatched patients are presented in Supplementary Table S3.

In the propensity score–matched cohort, the original Cox analysis showed significantly inferior overall survival in patients treated with definitive CRT compared with those treated with TL + PORT (median OS, 108 vs. 149 months; 5-year OS, 64.5% vs. 84.0%; HR, 2.08; 95% CI, 1.11–3.90; log-rank *p* = 0.020; Cox *p* = 0.023). After accounting for within-pair dependence using a cluster-robust variance estimator, overall survival remained significantly inferior in the definitive CRT group (HR, 2.08; 95% CI, 1.12–3.86; *p* = 0.021). Similarly, local control was significantly worse in the definitive CRT group (5-year LC, 77.7% vs. 100%; HR, 12.83; 95% CI, 2.76–59.55; log-rank *p* < 0.001; Cox *p* = 0.001). This association remained significant after accounting for within-pair dependence using a cluster-robust variance estimator (HR, 12.83; 95% CI, 3.75–43.87; *p* < 0.001). In contrast, disease-specific survival did not differ significantly between the definitive CRT and TL + PORT groups (5-year DSS, 87.7% vs. 96.5%; HR, 1.74; 95% CI, 0.53–5.66; log-rank *p* = 0.355). Similarly, distant metastasis-free survival was comparable between the groups (5-year DMFS, 94.2% vs. 92.6%; HR, 0.89; 95% CI, 0.20–3.98; log-rank *p* = 0.875) (Table 4). Furthermore, in the IPTW analysis including the entire cohort, overall survival did not differ significantly between the treatment groups (HR, 0.93; 95% CI, 0.64–1.36; *p* = 0.715).

### 3.4. Prognostic Factors

The potential prognostic impact of age, sex, smoking history, T stage (T3 vs. T4), N stage (N0 vs. N1), cumulative chemotherapy dose, tumor size, requirement for emergency tracheostomy, and, among surgically treated patients, surgical margin status, perineural invasion (PNI), and lymphovascular space invasion (LVSI) were evaluated.

No statistically significant prognostic factor associated with local control was identified in either treatment group.

Regarding distant metastasis, PNI and LVSI were identified as significant adverse prognostic factors in the TL + PORT group. In the definitive CRT group, patients with T4 tumors had a higher risk of distant metastasis compared with those with T3 tumors. Detailed results are presented in Table 5.

**Figure 3:**
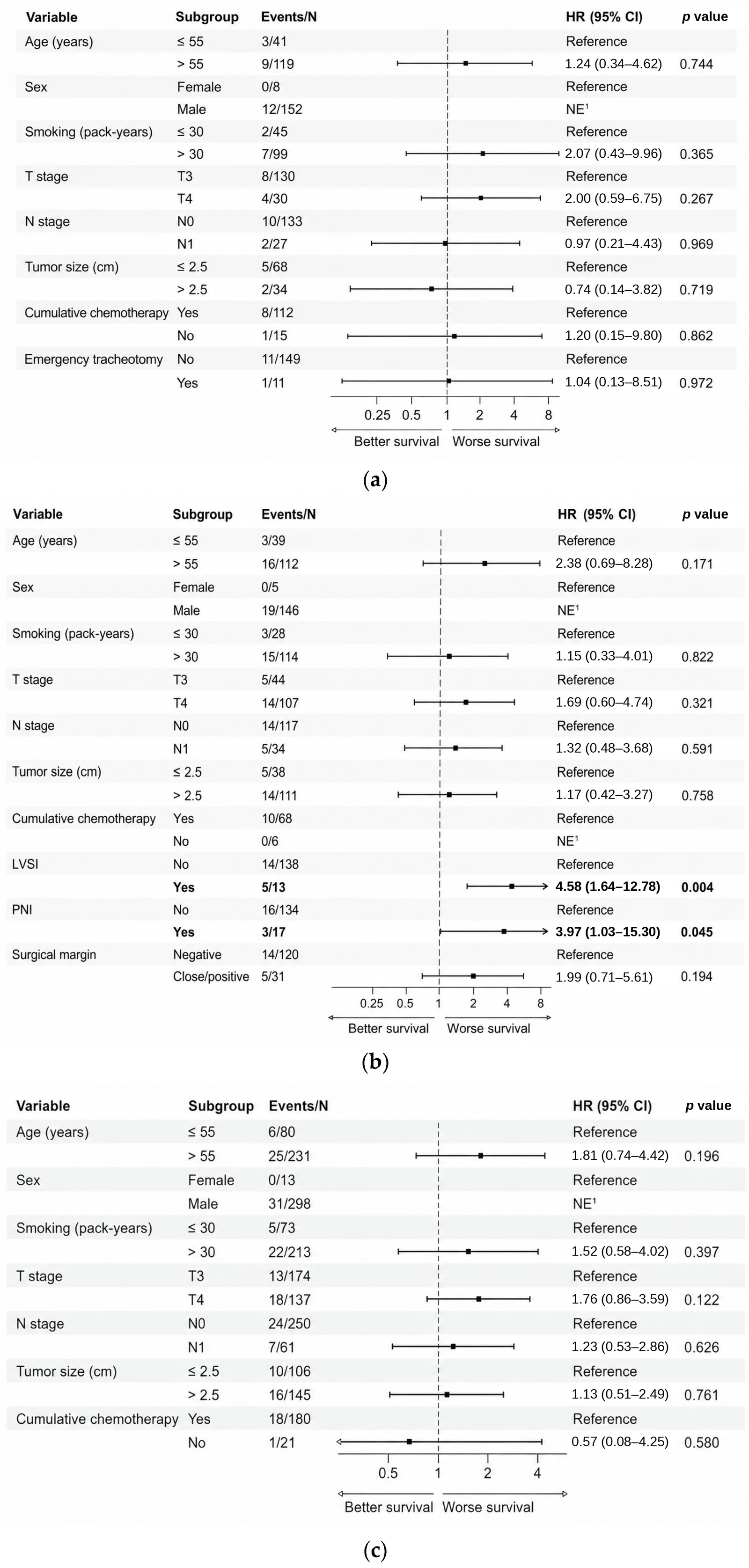
Forest plots demonstrating prognostic factors associated with disease-specific survival based on a univariable Cox proportional hazards regression analysis. (**a**) Definitive chemoradiotherapy group, (**b**) total laryngectomy with adjuvant radiotherapy/chemoradiotherapy group, and (**c**) entire cohort. ^1^ NE, not estimable because no events occurred in the corresponding comparison; LVSI, lymphovascular space invasion; PNI, perineural invasion.

For disease-specific survival, PNI and LVSI were identified as significant adverse prognostic factors in the TL + PORT group. In the definitive CRT group, T4 disease and lower cumulative chemotherapy exposure were associated with numerically inferior disease-specific survival, although neither association reached statistical significance. The results of the univariable Cox proportional hazards analyses are presented in the forest plots in Figure 3.

### 3.5. Functional Laryngeal Preservation and Laryngectomy-Free Survival

Functional laryngeal preservation was evaluated among patients treated with definitive CRT. During follow-up, two patients required permanent tracheostomy, both of whom had T3 disease. Four patients developed significant deterioration in swallowing function, including one patient who required long-term percutaneous endoscopic gastrostomy (PEG) support.

Among the 160 patients treated with definitive CRT, 31 (19.4%) developed local recurrence. Salvage total laryngectomy was performed in 20 patients, whereas 11 patients with local recurrence were managed without salvage laryngectomy. Among patients without local recurrence, 126 had no permanent functional sequelae, two had permanent tracheostomies, and one had long-term feeding-tube dependence. Using the prespecified definition, 126 of 160 patients (78.8%) achieved functional laryngeal preservation.

Laryngectomy-free survival was analyzed from the date of diagnosis, with total laryngectomy or death from any cause considered an event. The 5- and 10-year LFS rates were 74.6% and 62.3%, respectively.

In the competing-risk analysis, with death treated as a competing event, the cumulative incidence of salvage laryngectomy was 13.5% at 5 years and 14.9% at 10 years.

## 4. Discussion

In this multicenter retrospective cohort of patients with T3–T4a N0–N1 laryngeal cancer, definitive CRT was consistently associated with inferior local control compared with TL + PORT, whereas disease-specific survival remained comparable between treatment strategies. Overall survival estimates varied substantially across analytic approaches, with significant differences favoring TL + PORT in the propensity-matched and calendar-era/IMRT-restricted analyses, but no significant difference in the full-cohort IPTW analysis. Functional laryngeal preservation was achieved in 126 of 160 patients (78.8%) treated with definitive CRT. Taken together, these findings indicate a clear local-control advantage for surgery while supporting definitive CRT as an organ-preservation option in appropriately selected patients, without evidence of a stable treatment-specific effect on overall or disease-specific survival.

A distinctive feature of this study was the restriction of the cohort to patients with N0–N1 disease. Advanced nodal involvement is a major prognostic factor in locally advanced laryngeal cancer and may obscure the effect of the primary treatment strategy. Restricting the analysis to patients with limited nodal burden was therefore intended to reduce this source of clinical heterogeneity [30,31].

The overall survival findings varied substantially according to the analytic approach and should therefore be interpreted with caution. In the unmatched cohort, 10-year OS numerically favored definitive CRT (62.2% vs. 45.8% after TL + PORT), although the difference was not statistically significant (*p* = 0.088). In contrast, the propensity score–matched cohort showed inferior OS after definitive CRT, and this association remained significant after accounting for within-pair dependence using a cluster-robust variance estimator (HR, 2.08; 95% CI, 1.12–3.86; *p* = 0.021). The calendar-era and IMRT-restricted analysis similarly favored TL + PORT (HR, 3.64; 95% CI, 1.09–12.22; *p* = 0.036), although this estimate was based on only three OS events in the TL + PORT subgroup and should be interpreted cautiously. By contrast, the principal full-cohort IPTW analysis, which retained all 311 patients using multiple imputation, showed no significant OS difference (HR, 0.93; 95% CI, 0.64–1.36; *p* = 0.715). These divergent estimates likely reflect the combined effects of non-random treatment allocation, selection of a clinically distinct subset by PSM, sparse events in the contemporary era-restricted cohort, and residual confounding from unmeasured factors such as comorbidity. Furthermore, second primary malignancies occurred in 12% of patients in both treatment groups, predominantly lung cancer, indicating a substantial competing burden of morbidity and mortality unrelated to laryngeal cancer. Because detailed comorbidity data and a standardized comorbidity index were not available, OS cannot be interpreted as a reliable causal comparative endpoint in this retrospective dataset. In contrast, the consistent difference in local control across multiple analyses and the absence of a significant difference in disease-specific survival represent the more robust comparative findings of this study.

The observed pattern is broadly consistent with previous retrospective comparisons of larynx-preservation therapy and total laryngectomy, although direct comparisons are complicated by differences in patient selection and disease extent. Grover et al. [32], using the National Cancer Database, reported inferior overall survival among patients with T4a laryngeal squamous cell carcinoma treated with larynx-preservation CRT compared with total laryngectomy. Importantly, the CRT group in that study had more advanced nodal disease, illustrating the extent to which treatment-selection and disease-burden differences may influence comparative survival outcomes. Our restriction to N0–N1 disease was intended to minimize this source of confounding and may partly explain why OS estimates differed from those reported in less selected populations; nevertheless, the heterogeneity observed across our adjusted analyses indicates that residual confounding remains important.

The higher local recurrence rate observed after definitive CRT is also consistent with previous studies comparing surgery-based and organ-preservation approaches. Fuller et al. [16], in a large retrospective analysis of patients with T3 laryngeal cancer, reported higher locoregional control with surgery-based treatment than with definitive CRT (83% vs. 77%, respectively). However, salvage laryngectomy after local failure contributed to comparable ultimate locoregional control and survival outcomes. This observation is particularly relevant to our findings because, although definitive CRT was associated with substantially inferior local control, local recurrence occurred in only approximately 20% of patients, and salvage total laryngectomy was feasible in 20 of 31 patients with local recurrence. Among these patients, 15 (75%) were alive and disease-free at last follow-up. Thus, the higher incidence of local recurrence after definitive CRT did not uniformly translate into treatment failure at the level of long-term disease control.

This concept is consistent with the fundamental principle of organ-preservation strategies established by the Veterans Affairs Laryngeal Cancer Study [5] and RTOG 91-11 [7], in which concurrent CRT was shown to provide an effective approach for laryngeal preservation in appropriately selected patients. Our findings therefore support a treatment paradigm in which definitive CRT may be used to avoid upfront laryngectomy, provided that patients are carefully selected and followed closely, with timely salvage surgery available for persistent or recurrent disease. Importantly, our data also demonstrate that organ preservation should not be interpreted as equivalent to superior local tumor control, since the latter remained significantly better after TL + PORT.

Nevertheless, more cautious conclusions have been reported in studies focusing specifically on T4 disease. Gourin et al. [10] analyzed patients treated between 1985 and 2002 and reported that total laryngectomy provided superior locoregional control and overall survival compared with organ-preservation approaches in T4 laryngeal cancer. Importantly, that study also highlighted that anatomical preservation of the larynx does not necessarily translate into preservation of laryngeal function. The apparently more favorable functional outcomes in our cohort should therefore be interpreted in the context of differences in patient selection and treatment era. Our cohort was restricted to N0–N1 disease, all definitive CRT patients received IMRT, and contemporary imaging and multidisciplinary surveillance were routinely used.

The influence of treatment era and radiotherapy technique was specifically addressed in our sensitivity analysis. When both treatment groups were restricted to patients treated between 2015 and 2025 who received IMRT, the difference in local control remained significant. This finding is important because the local control advantage of TL + PORT could otherwise have been attributed to differences in radiotherapy technique or treatment era. The persistence of the local control difference in this contemporary IMRT-restricted cohort therefore strengthens the interpretation that the difference is related to the treatment strategy itself, although residual selection bias cannot be excluded.

Despite superior local control rates in the surgical group, disease-specific survival was comparable between treatment approaches in our study. The absence of a significant DSS difference in our cohort despite the marked difference in local control may reflect the effectiveness of salvage treatment after local recurrence, as well as the relatively low incidence of distant metastatic failure.

Additional analyses addressing the proportional hazards assumption and competing risks further strengthened the interpretation of the local control findings. Because the proportional hazards assumption was not fully satisfied for treatment in selected models, the 60-month restricted mean survival time (RMST) was used as a complementary measure. The RMST was shorter in the definitive CRT group for both OS and LC, with the larger difference observed for LC. Furthermore, competing-risk analyses treating death from any cause as a competing event demonstrated a substantially higher cumulative incidence of local recurrence after definitive CRT, whereas distant metastasis remained uncommon and did not show a consistent treatment-related disadvantage. These analyses provide additional support for the robustness of the local control difference while emphasizing the uncertainty surrounding treatment-related differences in OS.

Supporting this concept, Fuller et al. [16] reported improved locoregional control, disease-specific survival, and laryngectomy-free survival among patients treated with IMRT. Similarly, Nobacht et al. [33] demonstrated comparable disease-specific and overall survival outcomes between surgery and CRT in patients with T3–T4a laryngeal cancer treated with IMRT, with a 5-year laryngeal preservation rate of approximately 81%, which is consistent with our findings.

Accordingly, we reassessed functional laryngeal preservation using the entire definitive CRT cohort as the denominator rather than conditioning the analysis on patients who remained alive or free of local recurrence. Using this prespecified definition, 126 of 160 patients (78.8%) achieved functional laryngeal preservation. The resulting estimate is broadly consistent with the approximately 81% 5-year laryngeal preservation rate reported by Nobacht et al. [33], although direct numerical comparison should be made cautiously because endpoint definitions and study populations differ.

Laryngectomy-free survival provides an additional time-dependent measure of organ preservation that incorporates both laryngectomy and death. In our cohort, the 5- and 10-year LFS rates were 74.6% and 62.3%, respectively.

In the unmatched cohort, distant metastasis-free survival was numerically higher in the definitive CRT group; however, this difference did not reach statistical significance. In our cohort, the higher rate of distant metastasis in the TL + PORT group and the association of PNI and LVSI with metastatic risk highlight the importance of systemic disease control in locally advanced laryngeal cancer. However, given that the difference in DMFS did not reach statistical significance and the competing-risk sensitivity analysis did not demonstrate a consistent treatment-related difference, this numerical observation should not be interpreted as evidence of a definitive distant-control advantage for definitive CRT.

Our study has several strengths. First, the inclusion of only patients with N0–N1 disease reduced the confounding effect of advanced nodal disease and allowed a more homogeneous evaluation of treatment strategies in patients with advanced primary tumors. Second, all patients receiving definitive CRT were treated with modern IMRT techniques, and the long median follow-up duration of 98 months provides meaningful information regarding long-term oncologic and functional outcomes. The use of multiple complementary sensitivity analyses—including calendar-era/IMRT restriction, propensity score matching, cluster-robust matched-pair analysis, IPTW, RMST, and competing-risk analysis—further strengthens the assessment of the robustness of the treatment comparisons.

Several limitations should also be acknowledged. Due to the retrospective design, treatment allocation was not randomized and selection bias cannot be excluded. Patients undergoing TL + PORT had a higher proportion of T4 tumors, which may have influenced comparative outcomes. Propensity score matching substantially reduced measured baseline imbalance, but it could not account for unmeasured confounding and reduced the matched sample to 59 patients per treatment group. Moreover, comparison of matched and unmatched patients demonstrated that the matched cohort represented a selected subset of the original population. In particular, the matched definitive CRT cohort was enriched for T4 disease and for patients with recorded tumor-size measurements, whereas the matched TL + PORT cohort contained substantially fewer T4 tumors than the unmatched surgical cohort. The opposing shifts in 5-year OS between matched and unmatched patients in the two treatment groups further indicate that the PSM-derived OS estimate should be interpreted as subset-specific rather than as an average treatment effect applicable to the entire cohort. This concern is particularly relevant because tumor-size missingness was markedly differential between treatment groups. Accordingly, greater weight was given to the IPTW analysis, which retained the full cohort using multiple imputation, when interpreting the adjusted OS comparison. An additional limitation concerns the measurement of tumor size, which was included in the propensity score model but was not assessed using an identical method in the two treatment groups. Tumor size was based on pretreatment cross-sectional imaging in patients treated with definitive CRT, whereas pathological tumor size from the surgical specimen was used in patients undergoing TL + PORT. Although these measurements represent the best available tumor-size estimates for each treatment strategy, they are not directly equivalent and may have introduced measurement heterogeneity into the propensity score model. Consequently, residual confounding related to tumor burden cannot be excluded despite adjustment for tumor size. In particular, standardized patient-level data on pretreatment laryngeal function, vocal cord mobility, cartilage invasion, and other clinical factors influencing treatment selection were not consistently available. Although T stage was included in the adjustment models, it cannot fully capture these characteristics, and residual confounding therefore remains possible. Although surveillance strategies were similar between the treatment groups, the longer median follow-up in the TL + PORT group may have provided a greater opportunity for detecting late recurrences, which should be considered when interpreting the local control findings.

The proportional hazards assumption was formally assessed and was not fully satisfied for treatment in selected models. We therefore supplemented the Cox regression results with 60-month RMST estimates, which do not require the proportional hazards assumption. Although these analyses supported the robustness of the local control finding, competing events and unmeasured factors affecting treatment selection may still influence long-term outcome estimates.

Furthermore, the definitive CRT group was heterogeneous with respect to systemic therapy, including induction followed by concurrent treatment and the use of cisplatin, carboplatin, or cetuximab. Given the relatively small sizes of these subgroups, regimen-specific oncologic comparisons were not considered sufficiently robust and were therefore not performed. Emergency tracheostomy should be interpreted cautiously as a prognostic variable, as it may partly reflect underlying tumor burden and clinically significant airway compromise rather than an independent disease characteristic. Detailed surgery-specific morbidity outcomes, including pharyngocutaneous fistula, stomal stenosis, pharyngoesophageal stricture, voice rehabilitation status, and comprehensive swallowing outcomes, were not systematically recorded in the retrospective dataset and therefore could not be reliably analyzed. This limits a comprehensive comparison of treatment-related morbidity between the two treatment strategies. Furthermore, functional outcomes were retrospectively obtained from clinical records; therefore, patient-reported quality-of-life measures and standardized functional assessment tools were not available. Finally, although this study reflects the experience of two tertiary referral centers, the generalizability of the findings requires validation in prospective multicenter studies.

## 5. Conclusions

In this selected cohort of patients with T3–T4a laryngeal cancer and N0–N1 disease, definitive CRT was associated with higher rates of local recurrence and inferior local control compared with TL + PORT. This finding remained consistent across calendar-era/IMRT restriction, propensity score matching, cluster-robust matched-pair analysis, RMST, and competing-risk analysis. Overall survival estimates varied substantially across unmatched, propensity-matched, era-restricted, and IPTW analyses and therefore do not support a stable treatment-specific survival effect in this retrospective cohort, whereas disease-specific survival remained comparable between treatment strategies. Functional laryngeal preservation was achieved in 126 of 160 patients (78.8%) treated with definitive CRT when the entire definitive CRT cohort was used as the denominator. These findings support definitive CRT as an organ-preservation option in appropriately selected patients, while emphasizing careful patient selection, close surveillance, and timely salvage treatment for local recurrence.

## Figures and Tables

**Table 1:** Patient and treatment characteristics according to treatment group.

Variable	Category	Definitive CRT (*n* = 160)	TL and PORT (*n* = 151)
Age (range)	Median	61.7 (37–90)	61.0 (37–82)
Sex (%)	Female	8 (5)	5 (3.3)
	Male	152 (95)	146 (96.7)
Smoking history (%)	≤30 pack-years	45 (28.1)	28 (18.5)
	>30 pack-years	99 (61.9)	114 (75.5)
	Unknown	16 (10)	9 (6)
Alcohol use (%)	No	50 (31.3)	45 (29.8)
	Yes	52 (32.5)	75 (49.7)
	Unknown	58 (36.3)	31 (20.5)
T stage (%)	T3	130 (81.3)	44 (29.1)
	T4	30 (18.8)	107 (70.9)
N stage (%)	N0	133 (83.1)	117 (77.5)
	N1	27 (16.9)	34 (22.5)
Tumor subsite (%)	Supraglottic	21 (13)	47 (31)
	Glottic	56 (35)	45 (30)
	Transglottic	83 (52)	59 (39)
Chemotherapy (%)	No	5 (3.1)	74 (49)
	Yes	155 (96.9)	77 (51)
Timing of chemotherapy (%)	Induction and concurrent	23 (14.8)	0 (0)
	Concurrent only	132 (85.2)	77 (100)
Type of chemotherapy (%)	Weekly cisplatin	113 (72.9)	74 (96.1)
	Three-weekly cisplatin	14 (9)	0 (0)
	Carboplatin	10 (6.5)	1 (1.3)
	Cetuximab	18 (11.6)	2 (2.6)
Radiotherapy technique (%)	3D-CRT	0 (0)	86 (57.0)
	IMRT	160 (100)	65 (43.0)
Radiotherapy dose (range)	Median	69.7 (64–71)	61.3 (60–66)

Abbreviations:  CRT, chemoradiotherapy; 3D-CRT, three-dimensional conformal radiotherapy; IMRT, intensity-modulated radiotherapy; PORT, postoperative radiotherapy; TL, total laryngectomy.

**Table 2:** Survival outcomes in the calendar-era and IMRT-restricted sensitivity analysis.

Outcome	Definitive CRT (IMRT), *n* = 124	TL + PORT (IMRT), *n* = 54	HR (95% CI)	*p*-Value
**Overall survival (OS)**				
Median OS, months	NR	NR	3.64 (1.09–12.22)	0.036
1-year OS	99.1%	100.0%		
3-year OS	88.2%	98.1%		
5-year OS	83.5%	98.1%		
**Disease-specific survival (DSS)**				
Median DSS, months	NR	NR	1.38 (0.37–5.21)	0.634
1-year DSS	100.0%	100.0%		
3-year DSS	92.7%	98.1%		
5-year DSS	92.7%	98.1%		
**Local control (LC)**				
Median LC, months	NR	NR	11.36 (1.53–84.36)	0.017
1-year LC	91.0%	100.0%		
3-year LC	83.2%	100.0%		
5-year LC	80.5%	100.0%		
**Distant metastasis-free survival (DMFS)**				
Median DMFS, months	NR	NR	0.47 (0.10–2.35)	0.361
1-year DMFS	98.4%	98.1%		
3-year DMFS	97.4%	98.1%		
5-year DMFS	97.4%	92.2%		

Abbreviations: CRT, definitive chemoradiotherapy; TL + PORT, total laryngectomy followed by postoperative radiotherapy; IMRT, intensity-modulated radiotherapy; OS, overall survival; DSS, disease-specific survival; LC, local control; DMFS, distant metastasis-free survival; HR, hazard ratio; CI, confidence interval; NR, not reached. Note: The sensitivity analysis was restricted to patients treated between 2015 and 2025 who received IMRT in both treatment groups. Survival estimates were obtained using the Kaplan–Meier method. Hazard ratios were estimated using Cox proportional hazards regression; HR >1 indicates a higher event hazard in the definitive CRT group compared with the TL + PORT group. Additional information on follow-up duration, event counts, landmark confidence intervals, and numbers at risk is provided in Supplementary Table S1.

**Table 3:** Baseline characteristics before and after propensity score matching.

Variable	Before PSM Definitive CRT (*n* = 160)	Before PSM TL + PORT (*n* = 151)	Absolute SMD	After PSM Definitive CRT (*n* = 59)	After PSM TL + PORT (*n* = 59)	Absolute SMD
**Age (%)**						
≤55 years	41 (25.6)	39 (25.8)	0.005	12 (20.3)	12 (20.3)	0.000
>55 years	119 (74.4)	112 (74.2)		47 (79.7)	47 (79.7)	
**Smoking history (%)**						
≤30 pack-years	45 (28.1)	28 (18.5)		10 (16.9)	10 (16.9)	
>30 pack-years	99 (61.9)	114 (75.5)	0.266	44 (74.6)	43 (72.9)	0.009
Unknown	16 (10.0)	9 (6.0)		5 (8.5)	6 (10.2)	
**Sex (%)**						
Male	152 (95.0)	146 (96.7)	0.084	58 (98.3)	58 (98.3)	0.000
Female	8 (5.0)	5 (3.3)		1 (1.7)	1 (1.7)	
**T stage (%)**						
T3	130 (81.3)	44 (29.1)	1.226	34 (57.6)	32 (54.2)	0.068
T4	30 (18.8)	107 (70.9)		25 (42.4)	27 (45.8)	
**N stage (%)**						
N0	133 (83.1)	117 (77.5)	0.142	46 (78.0)	50 (84.7)	0.173
N1	27 (16.9)	34 (22.5)		13 (22.0)	9 (15.3)	
**Tumor size (%)**						
≤2.5 cm	68 (42.5)	38 (25.2)		22 (37.3)	24 (40.7)	
>2.5 cm	34 (21.3)	111 (73.5)	0.903	34 (57.6)	33 (55.9)	0.057
Unknown	58 (36.3)	2 (1.3)		3 (5.1)	2 (3.4)	

Abbreviations: PSM, propensity score matching; CRT, definitive chemoradiotherapy; TL + PORT, total laryngectomy followed by postoperative radiotherapy; SMD, standardized mean difference. Note: Data are presented as *n* (%). Absolute SMD values < 0.20 were considered indicative of acceptable covariate balance.

**Table 4:** Survival outcomes after propensity score matching and cluster-robust matched-pair sensitivity analysis.

Outcome	Definitive CRT (*n* = 59)	TL + PORT (*n* = 59)	Original PSM HR (95% CI)	Cluster-Robust HR (95% CI)	*p*-Value *
**Overall survival (OS)**					
Events, *n*	23	20			
Median OS, months	108	149	2.08 (1.11–3.90)	2.08 (1.12–3.86)	0.021
1-year OS	91.5%	98.3%			
3-year OS	72.4%	91.3%			
5-year OS	64.5%	84.0%			
**Disease-specific survival (DSS)**					
Events, *n*	6	6			
Median DSS, months	NR	NR	1.74 (0.53–5.66)	—	0.355
1-year DSS	100.0%	98.3%			
3-year DSS	90.5%	96.5%			
5-year DSS	87.7%	96.5%			
**Local control (LC)**					
Events, *n*	14	3			
Median LC, months	NR	NR	12.83 (2.76–59.55)	12.83 (3.75–43.87)	<0.001
1-year LC	82.6%	100.0%			
3-year LC	80.7%	100.0%			
5-year LC	77.7%	100.0%			
**Distant metastasis-free survival (DMFS)**					
Events, *n*	3	4			
Median DMFS, months	NR	NR	0.89 (0.20–3.98)	—	0.875
1-year DMFS	96.4%	96.6%			
3-year DMFS	94.2%	94.8%			
5-year DMFS	94.2%	92.6%			

Abbreviations: CRT, definitive chemoradiotherapy; TL + PORT, total laryngectomy followed by postoperative radiotherapy; OS, overall survival; DSS, disease-specific survival; LC, local control; DMFS, distant metastasis-free survival; HR, hazard ratio; CI, confidence interval; NR, not reached. * Original PSM estimates were obtained using conventional Cox proportional hazards regression. Matched-pair adjusted estimates were obtained using Cox proportional hazards models with a cluster-robust variance estimator based on matched-pair identifiers.

**Table 5:** Prognostic factors associated with local control and distant metastasis in patients treated with total laryngectomy plus postoperative radiotherapy (TL + PORT) or definitive chemoradiotherapy (CRT).

Prognostic Factor	TL and PORT (*n* = 151)	*p* Value	HR (95% CI)	Definitive CRT (*n* = 160)	*p* Value	HR (95% CI)
	No. of Events/Evaluable Patients (%)			No. of Events/Evaluable Patients (%)		
***Local control***						
**Age** ≤55 (Reference) vs. >55	3/39 (7.7%) vs. 4/112 (3.6%)	NS	0.60 (0.13–2.76)	9/41 (22.0%) vs. 22/119 (18.5%)	NS	0.89 (0.41–1.93)
**Sex** Female (Reference) vs. male	0/5 (0.0%) vs. 7/146 (4.8%)	–	NE	1/8 (12.5%) vs. 30/152 (19.7%)	NS	1.71 (0.23–12.55)
**Smoking history** ≤30 (Reference) vs. >30	1/28 (3.6%) vs. 6/114 (5.3%)	NS	1.49 (0.18–12.47)	8/45 (17.8%) vs. 18/99 (18.2%)	NS	1.06 (0.46–2.44)
**T stage** T3 (Reference) vs. T4	2/44 (4.5%) vs. 5/107 (4.7%)	NS	1.82 (0.35–9.54)	24/130 (18.5%) vs. 7/30 (23.3%)	NS	1.27 (0.55–2.95)
**N stage** N0 (Reference) vs. N1	4/117 (3.4%) vs. 3/34 (8.8%)	NS	2.21 (0.47–10.31)	27/133 (20.3%) vs. 4/27 (14.8%)	NS	0.68 (0.24–1.94)
**Emergency tracheostomy** No (Reference) vs. yes	7/122 (5.7%) vs. 0/29 (0.0%)	–	NE	28/149 (18.8%) vs. 3/11 (27.3%)	NS	1.57 (0.48–5.18)
**Tumor size** ≤2.5 cm (Reference) vs. >2.5 cm	3/38 (7.9%) vs. 4/111 (3.6%)	NS	0.60 (0.13–2.73)	12/68 (17.6%) vs. 8/34 (23.5%)	NS	1.35 (0.55–3.29)
**Cumulative chemotherapy** Yes (Reference) vs. no	6/68 (8.8%) vs. 0/6 (0.0%)	–	NE	22/112 (19.6%) vs. 3/15 (20.0%)	NS	1.13 (0.34–3.76)
**Surgical margin** Negative (Reference) vs. close/positive	6/120 (5.0%) vs. 1/31 (3.2%)	NS	1.26 (0.14–11.12)			
**LVSI** No (Reference) vs. yes	6/138 (4.3%) vs. 1/13 (7.7%)	NS	2.74 (0.32–23.56)			
**PNI** No (Reference) vs. yes	7/134 (5.2%) vs. 0/17 (0.0%)	NS	NE			
***Distant metastasis***						
**Age** ≤55 (Reference) vs. >55	1/39 (2.6%) vs. 14/112 (12.5%)	NS	5.29 (0.70–40.28)	2/41 (4.9%) vs. 4/119 (3.4%)	NS	0.78 (0.14–4.29)
**Sex** Female (Reference) vs. male	0/5 (0.0%) vs. 15/146 (10.3%)	–	NE	0/8 (0.0%) vs. 6/152 (3.9%)	–	NE
**Smoking history** ≤30 (Reference) vs. >30	2/28 (7.1%) vs. 12/114 (10.5%)	NS	1.58 (0.35–7.05)	1/45 (2.2%) vs. 4/99 (4.0%)	NS	2.08 (0.23–18.78)
**T stage** T3 (Reference) vs. T4	4/44 (9.1%) vs. 11/107 (10.3%)	NS	1.32 (0.42–4.18)	2/130 (1.5%) vs. 4/30 (13.3%)	0.019	7.80 (1.41–43.16)
**N stage** N0 (Reference) vs. N1	11/117 (9.4%) vs. 4/34 (11.8%)	NS	1.35 (0.43–4.23)	5/133 (3.8%) vs. 1/27 (3.7%)	NS	1.05 (0.12–9.03)
**Emergency tracheostomy** No (Reference) vs. yes	11/122 (9.0%) vs. 4/29 (13.8%)	NS	1.78 (0.57–5.60)	5/149 (3.4%) vs. 1/11 (9.1%)	NS	2.09 (0.23–19.22)
**Tumor size** ≤2.5 cm (Reference) vs. >2.5 cm	4/38 (10.5%) vs. 11/111 (9.9%)	NS	1.07 (0.34–3.36)	1/68 (1.5%) vs. 2/34 (5.9%)	NS	3.72 (0.34–41.09)
**Cumulative chemotherapy** Yes (Reference) vs. no	7/68 (10.3%) vs. 0/6 (0.0%)	–	NE	4/112 (3.6%) vs. 0/15 (0.0%)	–	NE
**Surgical margin** Negative (Reference) vs. close/positive	10/120 (8.3%) vs. 5/31 (16.1%)	NS	2.24 (0.76–6.58)			
**LVSI** No (Reference) vs. yes	11/138 (8.0%) vs. 4/13 (30.8%)	0.018	4.93 (1.56–15.59)			
**PNI** No (Reference) vs. yes	12/134 (9.0%) vs. 3/17 (17.6%)	0.042	3.69 (1.047–14.07)			

Abbreviations: CI, confidence interval; CRT, chemoradiotherapy; HR, hazard ratio; LVSI, lymphovascular space invasion; NE, not estimable because no events occurred in one of the comparison groups; NS, not significant; PNI, perineural invasion; PORT, postoperative radiotherapy; TL, total laryngectomy.

## Data Availability

The data presented in this study are available on request from the corresponding author. The data are not publicly available due to privacy and ethical restrictions.

## Supplementary Materials

The following supporting information can be downloaded at https://www.mdpi.com/article/10.3390/cancers18182966/s1, Supplementary Table S1: Detailed follow-up, event, and landmark estimates for overall survival and local control in the calendar-era and IMRT-restricted sensitivity cohort (2015–2025); Supplementary Table S2: RMST and competing-risk sensitivity analyses in patients treated with IMRT between 2015 and 2025; Supplementary Table S3: Baseline characteristics and crude outcomes of matched and unmatched patients within each treatment group.

## Abbreviations

The following abbreviations are used in this manuscript:

3D-CRT	Three-dimensional conformal radiotherapy
AJCC	American Joint Committee on Cancer
CI	Confidence interval
CRT	Chemoradiotherapy
CT	Computed tomography
DMFS	Distant metastasis-free survival
DSS	Disease-specific survival
HR	Hazard ratio
IGRT	Image-guided radiotherapy
IMRT	Intensity-modulated radiotherapy
IPTW	Inverse probability of treatment weighting
LC	Local control
LFS	Laryngectomy-free survival
LVSI	Lymphovascular space invasion
MRI	Magnetic resonance imaging
NR	Not reached
OS	Overall survival
PEG	Percutaneous endoscopic gastrostomy
PET/CT	Positron emission tomography/computed tomography
PNI	Perineural invasion
PORT	Postoperative radiotherapy
PSM	Propensity score matching
RC	Regional control
RMST	Restricted mean survival time
RT	Radiotherapy
SMD	Standardized mean difference
TL	Total laryngectomy
TL + PORT	Total laryngectomy followed by postoperative radiotherapy
VMAT	Volumetric-modulated arc therapy
