# Supplementary material for: Definitive Chemoradiotherapy Versus Total Laryngectomy Followed by Postoperative Therapy in Selected T3–T4a N0–N1 Laryngeal Cancer: Comparative Outcomes
Source: Cancers (Basel). 2026 Sep 14;18(18):2966. doi: 10.3390/cancers18182966 (PMC13604556; doi:10.3390/cancers18182966)
Supplement: Supplementary file 1 [file cancers-18-02966-s001.zip › cancers-4481044-supplementary.pdf]

Supplementary Table S1. Detailed follow-up, event, and landmark estimates for overall survival and local control in the calendar-era and IMRT-restricted sensitivity cohort (2015–2025).

Measure	Definitive CRT (IMRT), n=124	TL+PORT (IMRT), n=54
Median follow-up, months*	60	56
**Overall survival (OS)**		
OS events, n	21	3
1-year OS (95% CI)	99.1% (94.1–99.9%)	100.0%
Number at risk at 1 year, n	117	54
3-year OS (95% CI)	88.2% (80.5–93.0%)	98.1% (87.1–99.7%)
Number at risk at 3 years, n	86	47
5-year OS (95% CI)	83.5% (74.6–89.5%)	98.1% (87.1–99.7%)
Number at risk at 5 years, n	52	26
**Local control (LC)**		
Local recurrence events, n	22	1
1-year LC	91.0%	100.0%
3-year LC	83.2%	100.0%
5-year LC	80.5%	100.0%
Timing of the single TL+PORT local recurrence	—	92 months

*Median follow-up was estimated using the reverse Kaplan–Meier method.

Abbreviations: CRT, definitive chemoradiotherapy; TL+PORT, total laryngectomy followed by postoperative radiotherapy; IMRT, intensity-modulated radiotherapy; OS, overall survival; LC, local control; CI, confidence interval.

Note: The single local recurrence in the TL+PORT group occurred at 92 months, beyond the 5-year landmark. Accordingly, the 1-, 3-, and 5-year LC estimates remained 100%, although a local recurrence event was observed during the complete follow-up period and the Cox hazard ratio was therefore estimable. Only one OS event occurred in the TL+PORT group within the first 5 years; consequently, the 3- and 5-year OS estimates were both 98.1%. Landmark estimates should be interpreted in the context of the corresponding numbers at risk.

Supplementary Table S2. RMST and competing-risk sensitivity analyses in patients treated with IMRT between 2015 and 2025.

Outcome	Definitive CRT (n=124)	TL+PORT (n=54)	Between-group difference
**60-month RMST for OS**	54.6 months	59.4 months	−4.9 months
**60-month RMST for LC**	52.0 months	60.0 months	−7.9 months
**5-year cumulative incidence of local recurrence**	19.0%	0.0%	+19.0 percentage points
**5-year cumulative incidence of distant metastasis**	2.5%	7.8%	−5.2 percentage points

Abbreviations: CRT, chemoradiotherapy; TL+PORT, total laryngectomy followed by postoperative radiotherapy; IMRT, intensity-modulated radiotherapy; RMST, restricted mean survival time; OS, overall survival; LC, local control.

Note: The analysis was restricted to patients treated between 2015 and 2025 who received IMRT in both treatment groups. RMST was estimated up to 60 months. For competing-risk analyses, local recurrence and distant metastasis were treated as the event of interest and death as a competing event. Cumulative incidence functions were estimated using the Aalen–Johansen method.

Supplementary Table S3. Baseline characteristics and crude outcomes of matched and unmatched patients within each treatment group.

Characteristic/outcome	CRT matched n=59	CRT unmatched n=101	TL+PORT matched n=59	TL+PORT unmatched n=92
**Age >55 years**	47 (79.7%)	72 (71.3%)	47 (79.7%)	65 (70.7%)
**Male sex**	58 (98.3%)	94 (93.1%)	58 (98.3%)	88 (95.7%)
**Smoking >30 pack-years**	44 (74.6%)	55 (54.5%)	43 (72.9%)	71 (77.2%)
**Smoking unknown**	5 (8.5%)	11 (10.9%)	6 (10.2%)	3 (3.3%)
**T4 disease**	25 (42.4%)	5 (5.0%)	27 (45.8%)	80 (87.0%)
**N1 disease**	13 (22.0%)	14 (13.9%)	9 (15.3%)	25 (27.2%)
**Tumor size >2.5 cm**	34 (57.6%)	0 (0%)	33 (55.9%)	78 (84.8%)
**Tumor size unknown**	3 (5.1%)	55 (54.5%)	2 (3.4%)	0 (0%)
**OS deaths**	23 (39.0%)	26 (25.7%)	20 (33.9%)	54 (58.7%)
**5-year OS**	64.5%	81.7%	84.0%	63.5%
**DSS deaths**	6 (10.2%)	6 (5.9%)	6 (10.2%)	13 (14.1%)
**5-year DSS**	87.7%	94.5%	96.5%	91.7%
**Local recurrences**	14 (23.7%)	18 (17.8%)	3 (5.1%)	4 (4.3%)
**5-year LC**	77.7%	81.1%	100%	97.7%
**Distant metastases**	3 (5.1%)	3 (3.0%)	4 (6.8%)	11 (12.0%)
**5-year DMFS**	94.2%	97.9%	92.6%	89.4%

Abbreviations: CRT, definitive chemoradiotherapy; TL+PORT, total laryngectomy followed by postoperative radiotherapy; OS, overall survival; DSS, disease-specific survival; LC, local control; DMFS, distant metastasis-free survival.

Note: Data are presented as n (%) unless otherwise indicated. Five-year survival estimates were calculated using the Kaplan–Meier method.
